# Prosody Disorder and Sing-Song Speech in a Patient With Recurrent Glioblastoma: A Case Report

**DOI:** 10.7759/cureus.76385

**Published:** 2024-12-25

**Authors:** Narushi Sugii, Masahide Matsuda, Eiichi Ishikawa

**Affiliations:** 1 Department of Neurosurgery, University of Tsukuba, Tsukuba, JPN; 2 Department of Neurosurgery, University of Tsukuba Hospital, Tsukuba, JPN

**Keywords:** aphasia, dysprosody, glioblastoma, melodic intonation, sing-song speech

## Abstract

Dysprosody affects rhythm and intonation in speech, resulting in the impairment of emotional or attitude expression, and usually presents as a negative symptom resulting in a monotonous tone. We herein report a rare case of recurrent glioblastoma (GBM) with dysprosody featuring sing-song speech. A 68-year-old man, formerly left-handed, with right temporal GBM underwent gross total resection. After chemoradiation therapy, he was discharged without any deficits. Nineteen months later, the patient exhibited recurrence and presented a peculiar way of speaking with excessive melodic intonation. A head magnetic resonance imaging revealed new enhanced lesions in the residual right temporal lobe and the splenium of the corpus callosum with a massive surrounding T2-high area. The case highlights the bilateral hemispheric network underlying prosody and the compensatory failure caused by tumor progression and connectivity disruption. This first account of sing-song dysprosody in a GBM patient underscores the complexity of the language network and the need for further case accumulation to elucidate the pathophysiology of such rare presentations.

## Introduction

Human conversation consists of various factors, including interpersonal relationships and context, plus non-verbal and verbal communication [1]. Verbal communication itself encompasses meanings and mannerisms such as voice volume, speed, rhythm, stress, pause, and intonation. Prosody is a facet of verbal communication that comprises affective prosody, the expression of emotion in speech, and linguistic prosody, which is a specific focus on intonation [2]. Thus, dysprosody (prosody dysfunction) interferes with smooth conversation and language comprehension.

Dysprosody can be caused by various pathological conditions such as stroke [3], epilepsy [4], neurodegenerative diseases [5], and (theoretically) brain tumors [6]. However, to the best of our knowledge, no reports on brain tumors with dysprosody are currently published since patients with dysprosody generally show negative symptoms that lead to monotonous speech [4]. Conversely, there are no reports regarding positive symptoms of prosody disorders, especially in brain tumor patients.

Here, we report a case of recurrent glioblastoma (GBM) characterized by a sing-song intonation pattern and dysprosody.

## Case presentation

**Figure 1:**
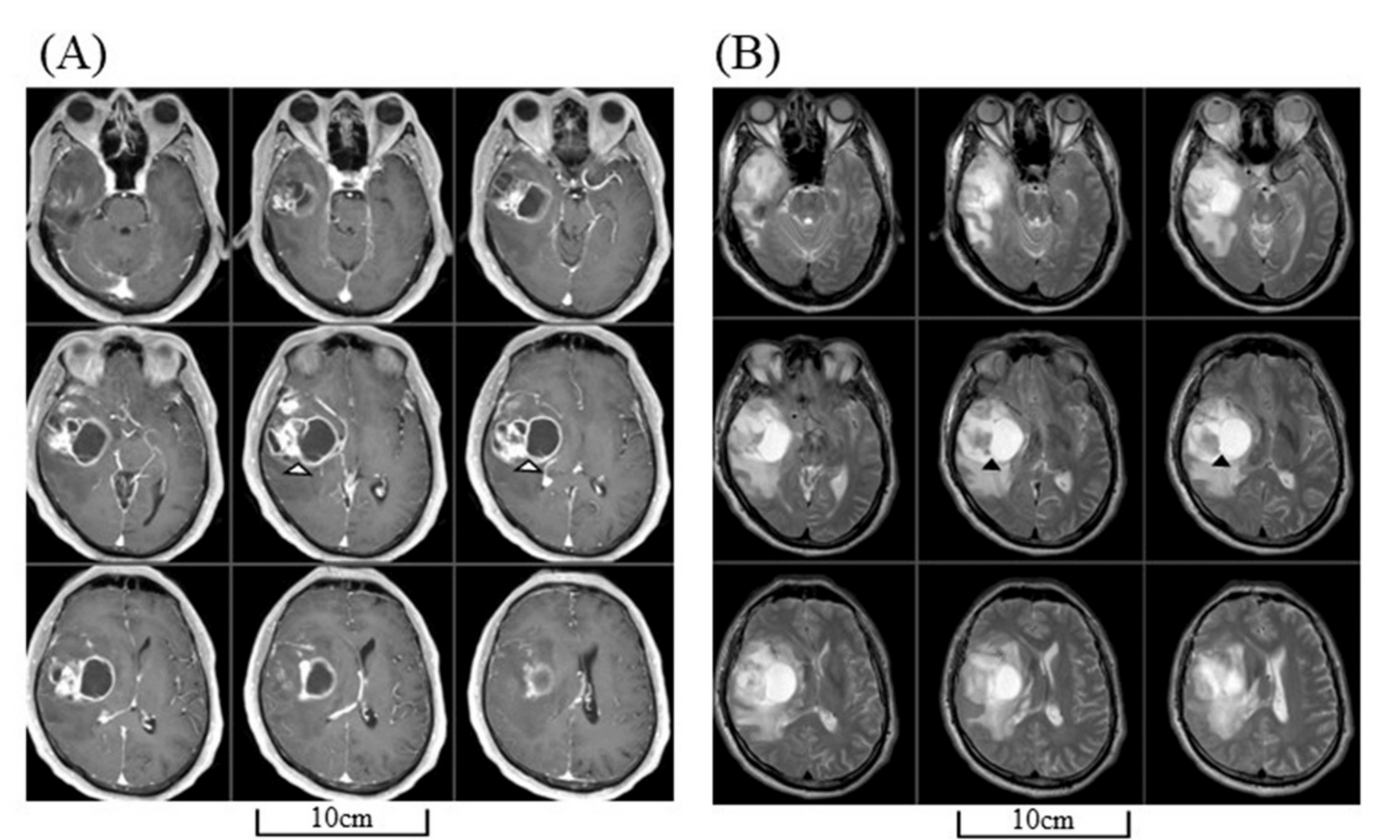
MRIs before surgery Head MRIs before surgery revealed a single mass lesion (50 mm in maximum diameter) located in the right temporal lobe (arrowheads). Post-enhanced T1-weighted images (A) and pre-enhanced T2-weighted images (B). MRI: magnetic resonance imaging

A formerly left-handed, 68-year-old man who had converted to right-handedness presented to our hospital complaining of headaches, mild left hemiparesis, and an increased tendency to be dazed at work. A head magnetic resonance imaging (MRI) revealed a 50 mm, sizeable single-mass lesion in the right temporal lobe, mainly in the superior temporal gyrus (STG) and middle temporal gyrus (MTG) (Figure 1).

**Figure 2:**
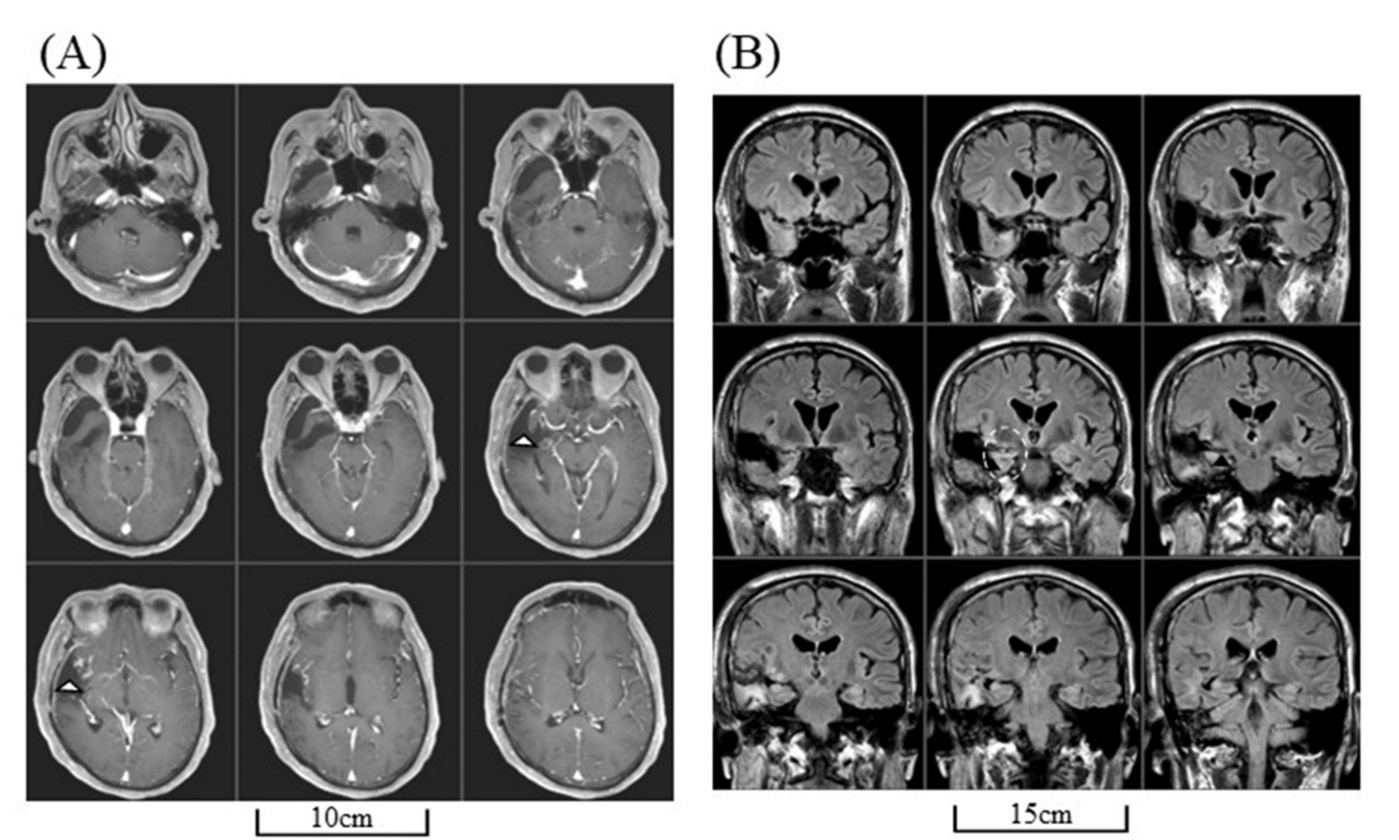
MRIs after initial treatment Head MRIs after initial treatments (i.e., surgery, chemotherapy with temozolomide, and radiation therapy) revealed no residual tumor in post-enhanced T1-weighted images (arrowheads) (A) and slight edema in the posterior part of the temporal lobe in FLAIR images (dotted circle) (B). FLAIR: fluid-attenuated inversion recovery; MRI: magnetic resonance imaging

The lesion showed ring-shaped contrast enhancement with surrounding edema. We suspected a high-grade glioma and performed gross total removal by resecting the ipsilateral uncus and anteroinferior part of the insular cortex along with the anterior STG and MTG (Figure 2).

**Figure 3:**
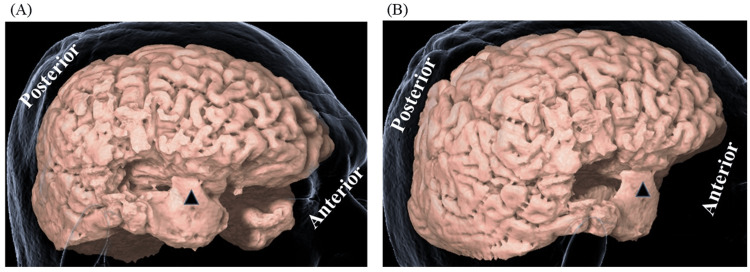
Three-dimensional (3D) brain images after initial treatment Three-dimensional (3D) brain images of lateral (A) and posterolateral (B) views are shown. Arrowheads indicate the preserved temporal tip.

We preserved the forward end of the STG and MTG but the surrounding fasciculus may have disconnected (Figure 3).

Although not evaluated using standardized assessment measures, such as the standard language test of aphasia (SLTA), aphasia did not occur after surgery and the patient was discharged without any symptoms. Since the pathological diagnosis at that time was GBM (isocitrate dehydrogenase (IDH)-wildtype, WHO grade IV), postoperative adjuvant therapy, including radiation therapy at 60 gray/30 fractions and chemotherapy with temozolomide, was conducted. Neuropsychological test results at the end of radiation therapy were as follows: mini-mental status examination (MMSE): 30/30; Hasegawa dementia scale-revised (HDS-R): 30/30; frontal assessment battery (FAB): 17/18; word recall of "ka": six words/minute; word recall of "vegetables": 13 words/minute; clock drawing test (CDT): 13/15; digit span (forward): six digits; figure copying: generally acceptable; and line bisection: normal.

Nineteen months later, the patient exhibited progressive memory loss, headaches, gait disturbance, mild left hemiparesis, and persistent prosody disorder with melodic intonation. He could speak nearly fluently with average volume but was slightly breathy and hoarse without articulatory distortion. He exhibited excessive intonation, repeating one note high and low at a time, and his speech sounded as if he was singing. He had mild-to-moderate impaired attention and disinhibition but could comply with instructions. His speech had a repetitive inflection with uncontrollable rhythm and melody that did not resemble extant ones. He had no significant paralysis of the tongue, face, or soft palate. SLTA was not performed but practical repetition, reading, and writing were generally possible. Neuropsychological test results showed an overall deterioration as follows: MMSE: 22/30; HDS-R: 21/30; FAB: 8/18; word recall of "ka": one word/minute; word recall of "vegetables": seven words/minute; CDT: 8/15; digit span (forward): six digits; figure copying: difficult; and line bisection: impaired with 12 mm to the right.

**Figure 4:**
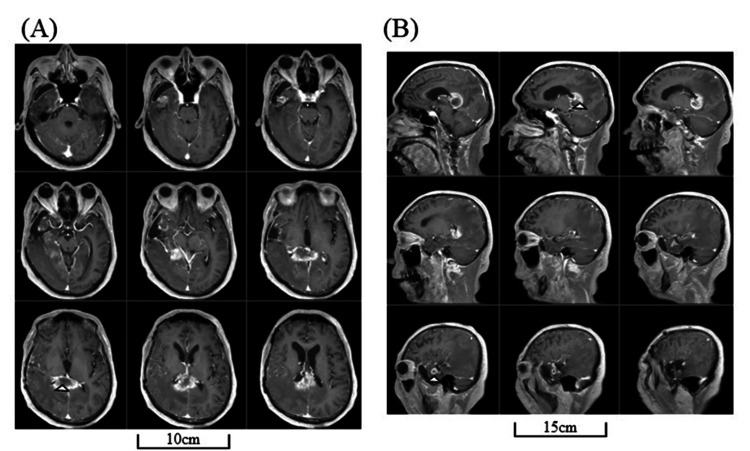
MRIs at recurrence Head MRIs at the recurrence revealing new enhanced lesions at the right residual anterior STG and MTG, hippocampus, and splenium of corpus callosum by axial (A) and sagittal (B) view of post-enhanced T1-weighted images (arrowheads). MRI: magnetic resonance imaging; MTG: middle temporal gyrus; STG: superior temporal gyrus

**Figure 5:**
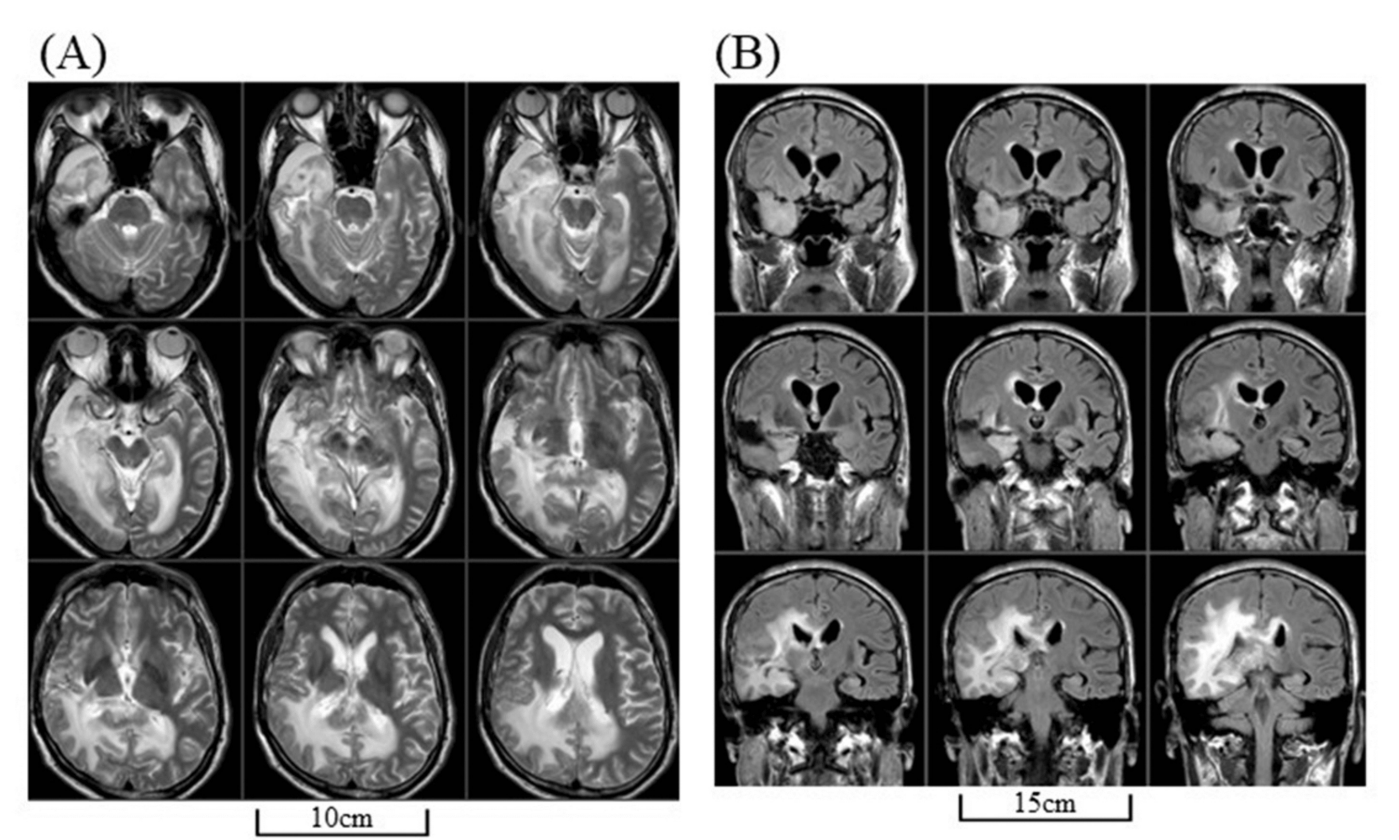
MRIs at recurrence (continued) The massive T2-high area extends even to the contralateral hemisphere by axial T2-weighted (A) and coronal FLAIR images (B). FLAIR: fluid-attenuated inversion recovery; MRI: magnetic resonance imaging

**Figure 6:**
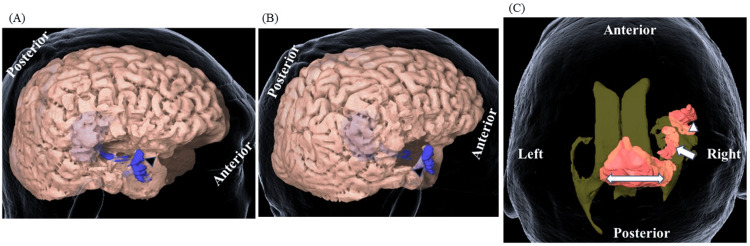
Three-dimensional (3D) brain images at recurrence Three-dimensional (3D) brain images of lateral (A) and posterolateral (B) views are shown. Dark-colored areas (marked by arrowheads) indicate the newly emerged enhanced lesions. A translucent 3D image showing ventricles and enhanced lesions at the temporal lobe (arrowhead), the hippocampus (arrow), and the splenium of the corpus callosum (double-headed arrow) (C).

An MRI revealed multiple, new, enhanced lesions in the residual anterior STG and MTG, hippocampus, and splenium of the corpus callosum (CC) with a massive surrounding T2-high area that extended to the contralateral parietal lobe (Figures 4-6).

We diagnosed a recurrence of GBM and administered bevacizumab as a second-line therapy. Nevertheless, his symptoms did not resolve over a month-long period and he unfortunately went into cardiopulmonary arrest due to pulmonary embolus two months after the recurrence. He was successfully resuscitated but remains severely disabled and was transferred to a long-stay hospital.

We obtained informed consent concerning the publication and handled the patient's clinical information anonymously in accordance with the principles of the Declaration of Helsinki and the "Act on the Protection of Personal Information" in Japan.

## Discussion

To the best of our knowledge, this is the first report of a recurrent GBM case with a peculiar symptom of sing-song speech. Although dysprosody can occur in various diseases [3-5], we assessed it as a recurrence of GBM based on the disease's progression and the imaging results. Dysprosody is a condition that affects rhythm and intonation in speech, resulting in the impairment of expressing one’s emotions or attitudes via conversations [7]. Generally, patients with dysprosody show negative symptoms, such as a loss of melody, in their speech [4,8]. However, the present case exhibited a positive symptom with melodic intonation; it is unique in this regard.

Historically, the right or non-dominant hemisphere, especially the right temporal lobe, is considered the center of prosody [4,9] but recent studies have revealed that other parts of the brain, including the left hemisphere, may be involved. Reported areas responsible for prosody are as follows: right STG and Heschl’s gyrus, bilateral anterior STGs (just anterior to Heschl’s gyrus), right posterior STG, bilateral mid posterior STGs, bilateral MTGs, right pars opercularis of inferior frontal gyrus (IFG), bilateral (left > right) pars orbitalis of IFGs, bilateral (right > left) middle (dorsal) precentral gyrus, right supplementary motor area (SMA) and pre-SMA, right medial prefrontal cortex, left supramarginal gyrus, right basal ganglia, right amygdala, and the right peri-Sylvian region [2,7,9-12]. Additionally, some articles reported the importance of associated fibers, such as the inferior longitudinal fasciculus (ILF) and inferior fronto-occipital fasciculus (IFOF), to prosody [13,14]. Thus, prosody is a function involving variable regions in “bilateral” cerebral hemispheres, which may add diverse information to language.

In the present case, new, enhanced lesions emerged in the residual anterior STG/MTG. However, it is doubtful that the preserved forward end of the STG/MTG impacted the language symptoms because the right temporal stem and surrounding area had been resected so circumjacent connecting fibers, such as the ILF, uncinate fasciculus, and anterior commissure, had already been lost. Besides, MRI at recurrence also revealed the enhanced lesion at the splenium of the CC. The CC is the most significant commissural fiber that connects cerebral hemispheres, and its splenium connects the right-and-left occipital, temporal, and parietal lobes. The functional integration of diverse and multiple sensory and cognitive processes necessary to generate contextual speech and tones relies entirely on these connections [15,16]. Thus, we believe that the CC lesion, plus the absence of already-lost fibers, brought a disruption of interhemispheric connections and a complete isolation of the right temporal lobe. Furthermore, the massive T2-high area that extended to the contralateral hemisphere most likely affected various other areas or fiber tracts that may have influenced the unique symptom. Our case, therefore, shows that prosody is processed by a network that mobilizes various regions of the bilateral cerebral hemispheres and the compensatory failure of this bi-spheric network resulted in atypical dysprosody with sing-song speech.

Although a lack of previous descriptions obscures the context of the unique symptom in this case, we speculate that multiple factors may have influenced it. One possible factor is the patient's change in hand dominance. He was once left-handed but shifted dominance to the right hand, especially for writing and chopstick use. Gliomas induce functional reorganization of the language area and left-handed people are more likely to suffer an atypical language effect, such as shifting of language lateralization to the right hemisphere and intra-hemispheric changes of representation, under such pathological conditions [17,18]. Although we did not perform the Wada test to determine language laterality, the absence of aphasia after radical resection of the tumor in the right hemisphere implied that the dominant hemisphere was left. Yet, we cannot deny that his speech area may have reorganized and resided bilaterally; if so, the atypical localization of language areas may have affected the unusual dysprosody.

Epileptic seizures were an important differential diagnosis to eliminate. It is true that up to 80% of GBM patients develop epileptic seizures during the disease [19] and epileptic seizures are likely to occur at the recurrence of high-grade gliomas [20]. However, we consider that the symptom was representative of a functional impairment caused by a compression or invasion of the tumor and surrounding edema since there were no suspected epileptic seizures and the symptom persisted over an entire month.

Singing differs from speaking and aphasic patients can sometimes sing words although they cannot speak [21]. Melodic intonation therapy is thus a speech rehabilitation tactic in patients with non-fluent aphasia to promote the involvement of “singing” in language processing [22]. In addition, there is a report of a low-grade glioma patient who automatically switched from speaking to singing language production mode by intraoperative stimulation at the pars opercularis in the right IFG during awake surgery [8]. Further accumulation of cases, although rare, is warranted to explore the pathologies associated with this unique presentation.

## Conclusions

We experienced a rare GBM case with a sing-song speech at recurrence. Although the specific area responsible for the symptom was unclear, prosody disorder with excessive intonation could be marked by a disruption of the language network in the bilateral cerebral hemispheres. This report underscores the complexity of the language network and the need for further case accumulation to elucidate the pathophysiology of such rare presentations.

## References

[1] Verderber KS, Sellnow DD, Verderber RF (2016). Communicate!, 16th Edition. https://www.cengageasia.com/title/default/detail?isbn=9780357799062.

[2] Belyk M, Brown S (2014). Perception of affective and linguistic prosody: an ALE meta-analysis of neuroimaging studies. Soc Cogn Affect Neurosci.

[3] Bodini B, Iacoboni M, Lenzi GL (2004). Acute stroke effects on emotions: an interpretation through the mirror system. Curr Opin Neurol.

[4] Peters AS, Rémi J, Vollmar C, Gonzalez-Victores JA, Cunha JP, Noachtar S (2011). Dysprosody during epileptic seizures lateralizes to the nondominant hemisphere. Neurology.

[5] Graff-Radford J, Jones DT, Graff-Radford NR (2014). Pathophysiology of language, speech and emotions in neurodegenerative disease. Parkinsonism Relat Disord.

[6] Sammler D, Cunitz K, Gierhan SM, Anwander A, Adermann J, Meixensberger J, Friederici AD (2018). White matter pathways for prosodic structure building: a case study. Brain Lang.

[7] Mitchell RL, Ross ED (2013). Attitudinal prosody: what we know and directions for future study. Neurosci Biobehav Rev.

[8] Herbet G, Lafargue G, Almairac F, Moritz-Gasser S, Bonnetblanc F, Duffau H (2015). Disrupting the right pars opercularis with electrical stimulation frees the song: case report. J Neurosurg.

[9] Hertrich I, Dietrich S, Ackermann H (2016). The role of the supplementary motor area for speech and language processing. Neurosci Biobehav Rev.

[10] Tierney A, Dick F, Deutsch D, Sereno M (2013). Speech versus song: multiple pitch-sensitive areas revealed by a naturally occurring musical illusion. Cereb Cortex.

[11] Cattaneo L (2013). Language. Handb Clin Neurol.

[12] Dichter BK, Breshears JD, Leonard MK, Chang EF (2018). The control of vocal pitch in human laryngeal motor cortex. Cell.

[13] Sihvonen AJ, Sammler D, Ripollés P, Leo V, Rodríguez-Fornells A, Soinila S, Särkämö T (2022). Right ventral stream damage underlies both poststroke aprosodia and amusia. Eur J Neurol.

[14] Schmidt AT, Hanten G, Li X (2013). Emotional prosody and diffusion tensor imaging in children after traumatic brain injury. Brain Inj.

[15] Edwards TJ, Sherr EH, Barkovich AJ, Richards LJ (2014). Clinical, genetic and imaging findings identify new causes for corpus callosum development syndromes. Brain.

[16] Palmer EE, Mowat D (2014). Agenesis of the corpus callosum: a clinical approach to diagnosis. Am J Med Genet C Semin Med Genet.

[17] Rösler J, Niraula B, Strack V (2014). Language mapping in healthy volunteers and brain tumor patients with a novel navigated TMS system: evidence of tumor-induced plasticity. Clin Neurophysiol.

[18] Ille S, Engel L, Albers L, Schroeder A, Kelm A, Meyer B, Krieg SM (2019). Functional reorganization of cortical language function in glioma patients-a preliminary study. Front Oncol.

[19] van Breemen MS, Rijsman RM, Taphoorn MJ, Walchenbach R, Zwinkels H, Vecht CJ (2009). Efficacy of anti-epileptic drugs in patients with gliomas and seizures. J Neurol.

[20] Di Bonaventura C, Albini M, D'Elia A (2017). Epileptic seizures heralding a relapse in high grade gliomas. Seizure.

[21] Racette A, Bard C, Peretz I (2006). Making non-fluent aphasics speak: sing along!. Brain.

[22] García-Casares N, Barros-Cano A, García-Arnés JA (2022). Melodic intonation therapy in post-stroke non-fluent aphasia and its effects on brain plasticity. J Clin Med.

